# Methanosuratincola petrocarbonis gen. nov., sp. nov., a methyl-reducing methanogen isolated from Shengli oil field, and proposal of Methanosuratincolaceae fam. nov., Methanosuratincolales ord. nov. and Methanosuratincolia classis nov. in the phylum Thermoproteota

**DOI:** 10.1099/ijsem.0.006839

**Published:** 2025-07-10

**Authors:** Kejia Wu, Lei Zhou, Fengfeng Zheng, Laiyan Liu, Min Yang, Jiang Li, Diana Z. Sousa, Lei Cheng

**Affiliations:** 1 Laboratory of Microbiology, Wageningen University and Research, Wageningen, The Netherlands; 2 Key Laboratory of Development and Application of Rural Renewable Energy, Biogas Institute of Ministry of Agriculture and Rural Affairs, Chengdu, PR China; 3 Shenzhen Key Laboratory of Marine Geo-Omics Research, Southern University of Science and Technology, Shenzhen, PR China

**Keywords:** ‘*Candidatus *Methanomethylicia’, methanogen, methyl-reducing methanogenesis, *Methanosuratincolia*, oil field

## Abstract

An anaerobic, thermophilic methanogen, designated strain LWZ-6^T^, was isolated from the Shengli oil field, China. The cells of strain LWZ-6^T^ were non-motile cocci, with a diameter of 0.5–1.0 µm, and formed aggregates. They reduced methanol and monomethylamine into methane, using H_2_ as an electron donor. Dimethylamine, trimethylamine and methanethiol, H_2_/CO_2_, formate, acetate, pyruvate, lactate and glucose were not used as energy sources. Strain LWZ-6^T^ required yeast extract, acetate or CO_2_ as carbon sources. Strain LWZ-6^T^ grew at 35–65 °C (optimum 55 °C), pH 5.0–8.0 (optimum 6.0–6.5) and 0–60 g l^−1^ NaCl (optimum 9 g l^−1^). The genome was 1.54 Mbp with a G+C content of 54.42 mol%. Strain LWZ-6^T^ shared 83.54% 16S rRNA gene sequence identity with *Infirmifilum lucidum* strain 3507LT^T^ in the class *Thermoprotei*. Phylogenetic analysis based on the 16S rRNA gene, along with phylogenomic analysis, indicated that strain LWZ-6^T^ belonged to the candidate class ‘*Candidatus* Methanomethylicia’, which lacks cultivated representatives. Based on these findings, a new species within a new genus, *Methanosuratincola petrocarbonis* gen. nov., sp. nov., is proposed for LWZ-6^T^ (=CCAM 1872^T^=JCM 39528^T^). In addition, we propose the *Methanosuratincolia* class. nov. for candidate class ‘*Ca*. Methanomethylicia’ represented by *Methanosuratincolaceae* fam. nov. and *Methanosuratincolales* ord. nov. within the phylum *Thermoproteota*.

## Introduction

Based upon the standardized archaeal taxonomy of the Genome Taxonomy Database (GTDB) [1] and the validly published names under the International Code of Nomenclature of Prokaryotes, all the cultured and isolated methanogens have been classified into the three phyla *Halobacteriota*, *Methanobacteriota* and *Thermoplasmatota* [2,4]. Members of ‘*Candidatus* Verstraetearchaeota’ have been proposed as methanogens based on their genomes, as they encode methyl-coenzyme M reductase, which catalyses the final step of methanogenesis, along with several methyltransferases potentially involved in methylotrophy [5,8]. Similarly, members in the phyla of ‘*Candidatus* Bathyarchaeota’ [9], ‘*Candidatus* Korarchaeota’ [10,11] and *Thaumarchaeota* [12] are speculated to engage in H_2_-dependent methylotrophic methanogenesis, reflecting the potential diversity of methanogens inferred from sequencing studies. These phyla ‘*Ca*. Bathyarchaeota’, ‘*Candidatus* Korachaeota’, ‘*Ca*. Verstraetearchaeota’ and *Thaumarchaeota* were later reclassified as the classes ‘*Candidatus* Bathyarchaeia’, ‘*Candidatus* Korarchaeia’, ‘*Candidatus* Methanomethylicia’ and *Nitrososphaeria*, respectively [1,13]. These potential methanogens are widely distributed in subsurface environments, including marine sediments, hot springs, wetlands and oil and gas fields [14], indicating an important role in global methane emissions and the global carbon cycle.

Recently, we successfully isolated strain LWZ-6^T^, the first methanogen in ‘*Ca*. Methanomethylicia’ of *Thermoproteota* from Shengli oil field, China. In a previous study, we detailed the isolation process of strain LWZ-6^T^. Strain LWZ-6^T^ was able to perform H_2_-dependent methylotrophic methanogenesis but did not show the fermentative metabolic capabilities that were previously speculated based on its genome [15]. In this paper, we formally describe the novel taxonomic lineage using the morphology, physiology and genomic data to propose the isolate into a novel genus *Methanosuratincola*. Furthermore, we propose a novel family, *Methanosuratincolaceae*, a novel order, *Methanosuratincolales*, and a novel class, *Methanosuratincolia* within the phylum *Thermoproteota*.

## Methods

### Isolation and cultivation

Samples for microbial isolation, including oily sludge and oil-produced water, were collected from Shengli oilfield in China (37°54′N, 118°33′E). The detailed composition of the samples and the isolation procedures for strain LWZ-6^T^ have been described previously [15]. Briefly, the isolation procedure consisted of a cocktail approach that combined traditional methods, such as serial dilution, gas exchange and antibiotic inhibition, with high-throughput cultivation using 96-well culture plate incubation. The purity of the isolate was confirmed by microscopy observation and PCR using the universal bacterial and archaeal primers [15]. The basal medium for strain LWZ-6^T^ contained (per litre) 9 g NaCl, 3 g MgCl_2_·6H_2_O, 0.15 g CaCl_2_·2H_2_O, 0.3 g NH_4_Cl, 0.2 g KH_2_PO_4_, 0.5 g KCl, 0.5 g l-cysteine-HCl, 1 ml 0.1% (w/v) resazurin solution and 2 ml trace element solution [16]. The basal medium was boiled for 30 min under 99.999% oxygen-free N_2_ and then transferred into serum bottles. The headspace of bottles was replaced with 99.999% oxygen-free N_2_. After autoclaving at 121 °C for 30 min, the basal medium was supplemented with 2 ml l^−1^ vitamin B_1_ solution (100 mg l^−1^ B_1_), 2 ml l^−1^ vitamin B_12_ solution (50 mg l^−1^ B_12_), 2 ml l^−1^ vitamin mixture [17] and 1% (v/v) selenite-tungstate (ST) solution [18]. In addition, solutions were added to give final concentrations in the basal medium of 10 mg l^−1^ 2-mercaptoethanesulfonic acid (CoM), 0.5 g l^−1^ casamino acids, 0.5 g l^−1^ yeast extract, 2 mM sodium acetate, 1 mM NaHCO_3_, 20 mM methanol and 10 kPa 100% H_2_. Finally, the medium was supplemented with 20 mM 2-morpholinoethanesulfonic acid monohydrate (MES) and was adjusted to pH 6.0–6.5 using 1 M HCl or NaOH solutions before use. The sterile stock solutions of vitamins, CoM, casamino acids, methanol and MES were made by filter-sterilizing, and sterile stock solutions of ST solution, yeast extract, sodium acetate and NaHCO_3_ were made by autoclaving. These stock solutions were all anaerobically prepared. All incubations were performed in triplicate in the dark without shaking.

### Microscopy

Cells were observed under an inverted fluorescence microscope (Eclipse 50i, Nikon). For scanning electronic micrographs, cells were fixed with 3% glutaraldehyde for 1 h at room temperature and washed with sterilized ultrapure water for 5 min twice. Following, samples were post-fixed in 1 % osmic acid for 2 h and washed with sterilized ultrapure water for 10 min three times. The fixed cells were dehydrated with 30%, 50%, 70%, 90% and 100% ethanol, each for 15 min. The dehydrated cells were sputter-coated with gold using a Smart Coater (JEOL, Tokyo, Japan) and observed under a JSM-IT710HR InTouchScope scanning electron microscope (JEOL, Tokyo, Japan).

### Phenotypic characterization

The potential of strain LWZ-6^T^ for H_2_-dependent methylotrophic methanogenesis was tested using different substrates, namely 10 mM methanol, 10 mM monomethylamine, 10 mM dimethylamine, 10 mM trimethylamine, 10 mM methanethiol and 10 mM betaine. 10 mM formate and 10 mM lactate were tested as alternative electron donors to H_2_ during methanol reduction. Additionally, the utilization of 10 mM formate, 20 kPa (4:1) H_2_/CO_2_ and 10 mM acetate as sole energy and carbon sources for methanogenesis was also tested. Fermentative capabilities of strain LWZ-6^T^ were tested using 10 mM lactate, 10 mM pyruvate, 10 mM glucose and 0.5 g l^−1^ casamino acids.

The following growth factors were tested in basal medium with methanol and H_2_ headspace: 10 mM acetate, 0.5 g l^−1^ casamino acids, 10 mg l^−1^ CoM, 10 mM NaHCO_3_, 1% (v/v) ST solution, 2 ml l^−1^ Wolin vitamin mixtures and 0.5 g l^−1^ yeast extract, each added separately. The carbon sources were tested in basal medium with methanol and H_2_ in headspace by adding ^13^C-acetate, ^13^C-NaHCO_3_ and ^13^C-yeast extract. The labelled lipids of strain LWZ-6^T^ were determined and analysed as previously described [15].

The temperature range for the growth of strain LWZ-6^T^ was determined by growing cultures at 25, 35, 40, 45, 50, 55, 60, 65 and 75 °C with pH at 6.0–6.5 and with 9 g l^−1^ NaCl. The pH range for the growth of strain LWZ-6^T^ was determined from pH 4 to pH 9, with a 0.5 increment, and at 55 °C with 9 g l^−1^ NaCl. The salinity range for the growth of strain LWZ-6^T^ was determined at 0, 9, 20, 30, 40, 50, 60 and 70 g l^−1^ NaCl, at pH 6.0–6.5 and 55 °C. The O_2_ tolerance of strain LWZ-6^T^ was determined by adding 1%, 2%, 5%, 10% and 20% oxygen (O_2_:N_2_, v/v) in the headspace. All incubations were performed in triplicate in the dark without shaking. CH_4_ and CO_2_ were analysed with a gas chromatograph (Agilent GC 7820A) as previously described [15].

### Lipid extraction and analysis

Cells of strain LWZ-6^T^ were harvested in the mid-to-late log phase for lipid analysis. Core membrane lipids were extracted using a modified acid hydrolysis method [19], and intact polar lipids were extracted using a modified Bligh and Dyer method [20]. The core and intact polar lipids were analysed using a Waters ACQUITY I-Class ultra-performance liquid chromatography coupled to a SYNAPT G2-Si quadrupole time-of-flight high-resolution mass spectrometer through an electrospray ionization source as previously described [15]. The raw data were converted to mzML format and processed with MS-DIAL software (v4.9.2). An in-house *in silico* MSMS spectral library of archaeal lipids was used for lipid identification.

### Genome sequencing and phylogenetic analysis

The complete genome sequence of strain LWZ-6^T^ was previously sequenced and annotated [15]. Maximum likelihood (ML) trees based on 16S rRNA and *mcrA* genes were reconstructed using IQ-TREE v2.3.6 (-bb 1000 -alrt 1000) with an optimal model selected by ModelFinder (-m MFP), using draft genomes of microorganisms within the phyla *Halobacteriota*, *Methanobacteriota*, *Thermoplasmatota* and *Thermoproteota* (with ≥75% completeness and ≤5% contamination, CheckM v1.0.8) obtained from publicly available databases at GTDB, Joint Genome Institute (JGI) and National Center for Biotechnology Information (NCBI). The best-fit model of the 16S rRNA gene was GTR+F+I+R3, and the *mcrA* gene was LG+C40+F+G as chosen by the Bayesian information criterion [21]. Prior to tree reconstruction, the 16S rRNA gene sequences were aligned and masked against reference archaeal 16S rRNA gene sequences using SSU-ALIGN (v.0.1.1), and the *mcrA* gene sequences were aligned using MAFFT (v7.526). Columns with gaps in more than 50% of the sequences were removed using trimAl (v1.5.rev0).

In addition to the ML trees based on 16S rRNA and *mcrA* genes, a phylogenetic tree was reconstructed using a set of 76 archaeal marker proteins (Archaea 76) [22]. Alignment was run with GToTree (v1.7.07), and columns with gaps in more than 90% of the sequences were removed using trimAl (v1.5.rev0). The ML tree was constructed using IQ-TREE v2.3.6 (-bb 1000 -alrt 1000) with the model LG+C60+F+G. Taxonomic classification of MAGs was performed according to the GTDB database (release 220, April 2024) [23].

The 16S rRNA gene identities between LWZ-6^T^ and related strains were calculated by blastn. The average nucleotide identity (ANI) was calculated using OrthoANIu (Orthologous ANI using USEARCH) tool [24] and the average amino acid identity (AAI) was calculated using CompareM (v.0.1.2; https://github.com/dparks1134/CompareM).

The 16S rRNA gene of strain LWZ-6^T^ was submitted to the Integrated Microbial Next Generation Sequencing (IMNGS) platform (https://www.imngs.org/). A similarity cut-off of 97% against the 16S rRNA gene at a minimum length of 200 bp was retrieved in the Sequence Read Archive using IMNGS for the environmental distribution analysis.

## Results and discussion

### Cell morphology

**Fig. 1.**
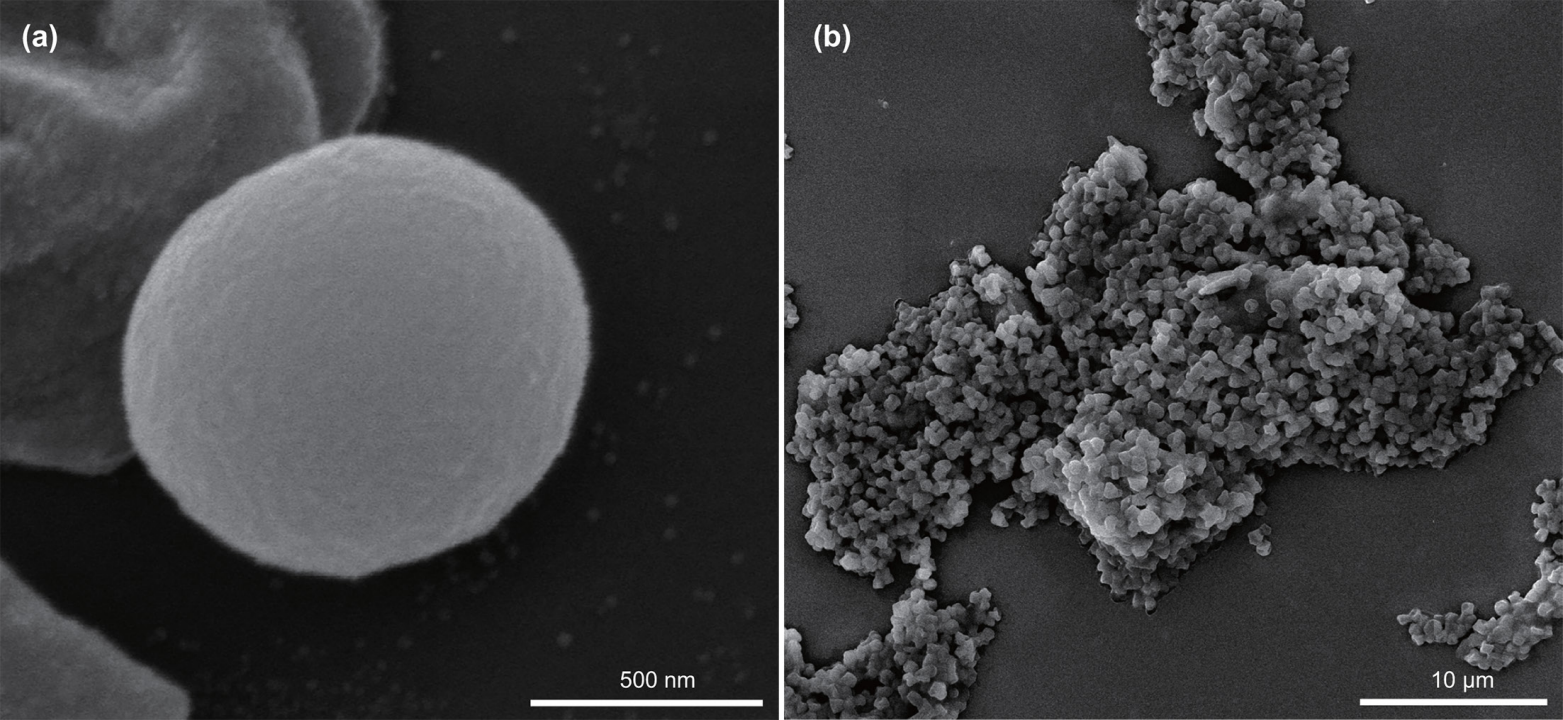
Scanning electron micrographs of strain LWZ-6^T^. Strain LWZ-6^T^ was cultured in fresh medium with methanol and H_2_ as substrates at 55 °C for 20 days. Scale bars are shown in the figure.

Cells of strain LWZ-6^T^ were non-motile, cocci-shaped with 0.5–1.0 µm diameter and formed aggregates during exponential growth (Fig. 1). Cells lysed in the presence of SDS (0.01%, w/v). No fluorescence at 420 nm could be observed for strain LWZ-6^T^.

### Physiology

Strain LWZ-6^T^ is a strict anaerobe, failing to grow in the presence of 1% (v/v) O_2_ (data not shown). It uses methylated compounds as electron acceptors and H_2_ as an electron donor to produce methane. The methylated compounds used for methanogenesis were methanol and monomethylamine, but not dimethylamine, trimethylamine, betaine and methanethiol. Formate and lactate did not serve as electron donors to reduce methylated compounds. Formate, H_2_/CO_2_ and acetate were not used for methanogenesis (Table S1, available in the online Supplementary Material).

*Ca*. Methanomethylicia was speculated to perform sugar, peptides and amino acids degradation based on the presence of genes for glycolysis and amino acid utilization [5,7]. We tested the fermentative metabolism of LWZ-6^T^, but no growth was observed in the presence of glucose, pyruvate, acetate and casamino acids. Therefore, it is likely that genes associated with glycolysis and amino acid utilization are involved in biosynthetic routes, i.e. gluconeogenesis and amino acid biosynthesis [15].

Yeast extract, acetate and CO_2_ were used as carbon sources for strain LWZ-6^T^ [15]. Yeast extract was an essential growth factor for strain LWZ-6^T^. Strain LWZ-6^T^ could grow between 35 and 65 °C, with an optimum temperature of 55 °C. Strain LWZ-6^T^ was slightly acidophilic, and the pH range for growth was between pH 5.0 and pH 8.0, with an optimal value at pH 6.0–6.5. Strain LWZ-6^T^ could tolerate NaCl concentration to 60 g l^−1^, with growth from 0 g l^−1^ to 40 g l^−1^ NaCl at an optimum of 9 g l^−1^ NaCl (Fig. S1).

### Chemotaxonomy

The core membrane lipids of strain LWZ-6^T^ were composed of archaeol (diphytanyl glycerol diether), a mixture of glycerol dibiphytanyl glycerol tetraethers (GDGTs) and glycerol monoalkyl glycerol tetraethers (GMGTs). The diether lipids were dominated by archaeol (20:0_20:0). The tetraether lipids consisted of GDGTs with 0 to 4 cyclopentane (GDGTs 0–4), GMGTs with up to two cyclopentane moieties and GMGTs with one additional methyl in the core structure (Homo-GMGTs 0–2). The dominant headgroups of intact polar lipids were one to four glycosidic, phosphatidylglycerol, glycosidic-phosphatidylglycerol and two glycosidic-phosphatidylglycerol moieties.

### Genomic features and phylogeny

**Fig. 2.**
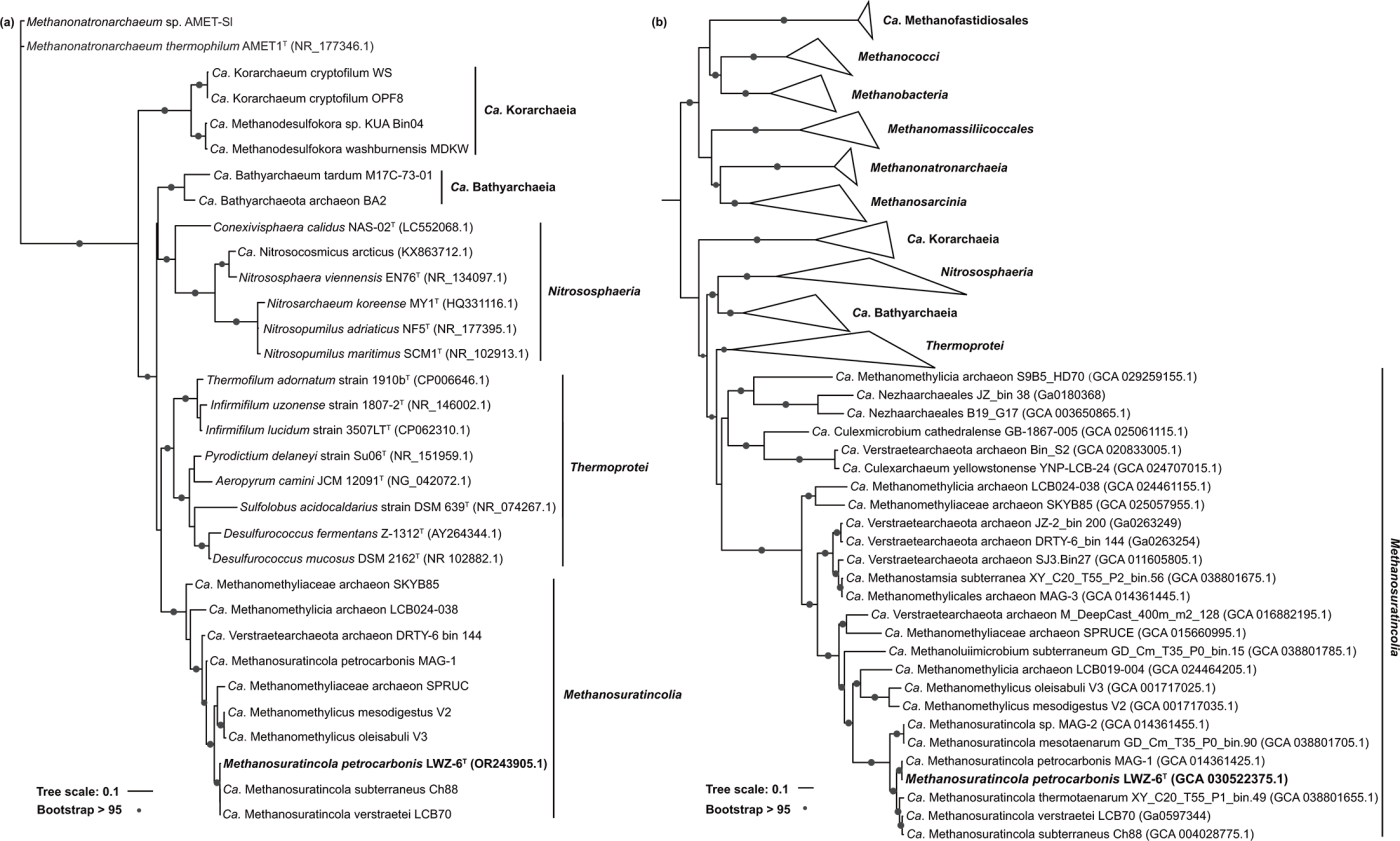
ML trees showing the phylogenetic position of strain LWZ-6^T^ (bold) with *Methanosuratincolia* members and other related strains in *Thermoproteota*, based on (**a**) 16S rRNA gene and (**b**) conserved archaeal marker proteins. *Methanonatronarchaeum* was chosen as an outgroup for the 16S rRNA gene-based comparison. Bootstrap values (>95%) are indicated by circles.

**Fig. 3.**
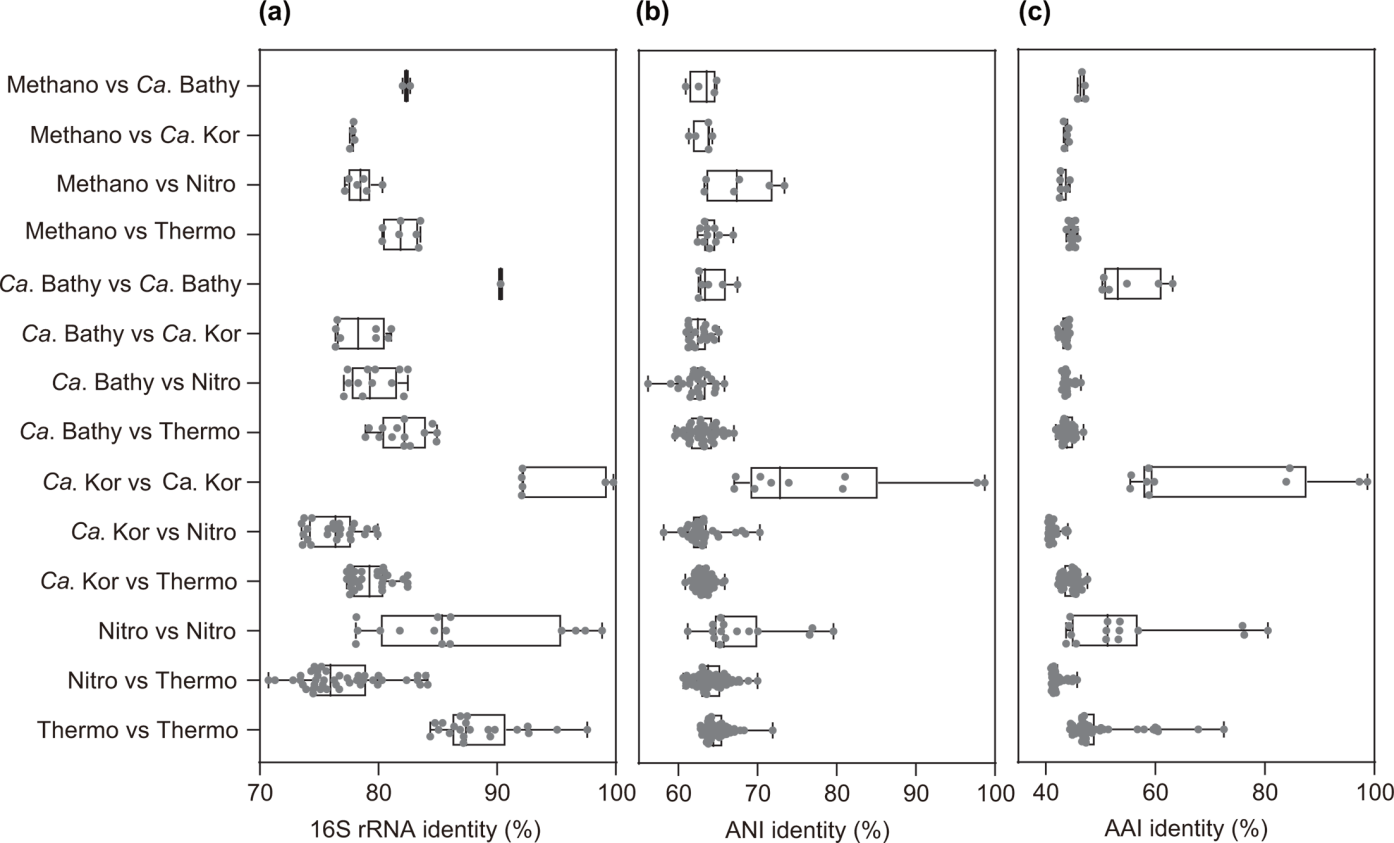
Comparison of (**a**) 16S rRNA gene identity, (**b**) ANI and (**c**) AAI within and between different phylogenetic groups. Methano: strain LWZ-6^T^, *Ca*. Bathy, *Ca*. Bathyarchaeia; *Ca*. Kor, *Ca*. Korarchaeia; Nitro, *Nitrososphaeria*; Thermo: *Thermoprotei*.

The genome size of strain LWZ-6^T^ was 1.54 Mbp with a DNA G+C content of 54.42 mol%. The genome encoded 1,606 predicted protein genes; one copy of each 5S, 16S and 23S rRNA genes; and 45 tRNAs. Strain LWZ-6^T^ shared 16S rRNA gene similarities of 99.67% with *Ca*. Methanosuratincola verstraetei LCB-70 and 99.60% with *Ca*. Methanosuratincola subterraneus Ch88, which formed an independent cluster based on phylogenetic analysis (Fig. 2a). Strain LWZ-6^T^ shared an ANI of 87.62% with *Ca*. Methanosuratincola verstraetei LCB-70 and 87.07% with *Ca*. Methanosuratincola subterraneus Ch88, demonstrating strain LWZ-6 represents a different species in *Ca*. Methanosuratincola (Tables S2 and S3). Strain LWZ-6^T^ coupled with the *Ca*. Methanosuratincola species shared 16S rRNA gene similarities, ANI and AAI with *Ca*. Methanomethylicus of 94.72–95.06%, 68.50–68.90% and 62.50–62.84%, respectively, demonstrating *Ca*. Methanosuratincola and *Ca*. Methanomethylicus are distinct genera in ‘*Ca*. Methanomethylicia’ (Fig. 2, Table S2–S4). Considering cultivated strains, strain LWZ-6^T^ shared the highest 16S rRNA gene sequence identity (83.54%) with *Infirmifilum lucidum* strain 3507LT^T^ in the class *Thermoprotei* in the phylum *Thermoproteota*. The 16S rRNA gene identities between strain LWZ-6^T^ and other isolated related strains within *Thermoproteota* ranged from 77.17% to 83.54% (Fig. 3). The phylogenomic tree and phylogeny tree based on 16S rRNA gene and *mcrA* gene (Figs 2 and S2) revealed that strain LWZ-6^T^ belongs to ‘*Ca*. Methanomethylicia’, forming a distinct cluster apart from four other classes in *Thermoproteota*. The ANI and AAI values between LWZ-6^T^ and the strains of the other four related classes in *Thermoproteota* (‘*Ca*. Bathyarchaeia’ and ‘*Ca*. Korachaeia’, *Nitrospheaeria* and *Thermoprotei*) were below 75% [25,26], demonstrating that LWZ-6^T^ represents a different class (Tables S3 and S4).

### Ecology

Metagenomic screening of the global distribution of LWZ-6^T^-related populations indicated their presence in diverse natural anoxic environments, including freshwater and sediments, marine sediments, hot springs, petroleum fields, soda sediments, soil and gut (Table S5). Additionally, they were found in man-made anoxic environments, such as anaerobic digesters. Together with evidence of the abundance of other H_2_-dependent methylotrophic methanogens, which are widespread in diverse anoxic environments [27,32], these findings suggest that this methanogenic pathway may play a significant role in global methane emissions.

Based on the phenotypic, genotypic and phylogenetic characteristics, we propose the novel methyl-reducing methanogen from an oil field as a new species of a new genus, for which the name *Methanosuratincola petrocarbonis* gen. nov., sp. nov. is proposed. In addition, based on phylogenetic analysis, we further propose a novel family, *Methanosuratincolaceae*, a novel order, *Methanosuratincolales*, and propose *Ca*. Methanomethylicia as a new class *Methanosuratincolia* classis nov. within the phylum *Thermoproteota*.

## Description

### Description of *Methanosuratincola* gen. nov.

*Methanosuratincola* (Me.tha.no.su.rat.in’co.la. N.L. pref. *methano-*, pertaining to methane; L. masc./fem. n. *incola,* inhabitant, dweller; N.L. masc. n. *Methanosuratincola*, methane organism inhabiting the Surat Basin).

Anaerobic, non-motile, coccid, neutrophilic and thermophilic methanogen. Utilize methanol and monomethylamine as electron acceptors, H_2_ as an electron donor to produce methane. Yeast extract is an essential growth factor. The carbon sources are yeast extract, acetate and CO_2_. The core membrane lipids are composed of archaeol, a mixture of GDGTs and GMGTs. The genome size is 1.54 Mbp, and the DNA G+C content is 54.42 mol%. The type species is *M. petrocarbonis*.

### Description of *Methanosuratincola petrocarbonis* sp. nov.

*petrocarbonis* (pe.tro.car.bo’nis. Gr. fem. n. *petra*, rock; L. masc. n. *carbo*, coal; N.L. gen. n. *petrocarbonis*, of coal from a rock).

Cells are non-motile and coccoid with a diameter of ~0.5–1.0 µm. Grows anaerobically and reduces methanol and monomethylamine into methane, using H_2_ as an electron donor. Dimethylamine, trimethylamine and methanethiol, H_2_/CO_2_, formate, acetate, pyruvate, lactate and glucose are not used as energy sources. Yeast extract is an essential growth factor. The carbon sources are yeast extract, acetate and CO_2_. Grows between 35 and 65 °C (optimum at 55 °C), pH ranges from 5.0 to 8.0 (optimum at pH 6.0–6.5) and between 0 and 60 g l^−1^ NaCl (optimum at 9 g l^−1^). The core membrane lipids are composed of archaeol, a mixture of GDGTs and GMGTs. The genome size is 1.54 Mbp, and the DNA G+C content is 54.42 mol%.

The type strain, LWZ-6^T^ (=CCAM 1872^T^=JCM 39528^T^), was isolated from oily sludge of the Shengli oil field in China.

The GenBank accession and assembly numbers of 16S rRNA and genome are OR243905 and GCA_030522375.1, respectively.

### Description of *Methanosuratincolaceae* fam. nov.

*Methanosuratincolaceae* (Me.tha.no.su.rat.in.co.la.ce’ae N.L. masc. n. *Methanosuratincola*, type genus of the family; -*aceae*, ending to designate a family; N.L. fem. pl. n. *Methanosuratincolaceae*, the *Methanosuratincola* family).

Description of the family *Methanosuratincolaceae* is the same as the genus *Methanosuratincola*. The type genus is *Methanosuratincola*.

### Description of *Methanosuratincolales* ord. nov.

*Methanosuratincolales* (Me.tha.no.su.rat.in.co.la’les. N.L. masc. n. *Methanosuratincola*, type genus of the order; -*ales*, ending to designate an order; N.L. fem. pl. n. *Methanosuratincolales*, the *Methanosuratincola* order).

Description of the order *Methanosuratincolales* is the same as the genus *Methanosuratincola*. The type genus is *Methanosuratincola*.

### Description of *Methanosuratincolia* classis nov.

*Methanosuratincolia* (Me.tha.no.su.rat.in.co’li.a. N.L. masc. n. *Methanosuratincola*, type genus of the class; -*ia*, ending to designate a class; N.L. neut. pl. n. *Methanosuratincolia*, the *Methanosuratincola* class).

Description of the class *Methanosuratincolia* is the same as the genus *Methanosuratincola*. The type genus is *Methanosuratincola*.

## Supplementary material

10.1099/ijsem.0.006839 Uncited Supplementary Material 1.

10.1099/ijsem.0.006839 Uncited Supplementary Material 2.

## Abbreviations

AAI: average amino acid identity
ANI: average nucleotide identity
GDGTs: glycerol dibiphytanyl glycerol tetraethers
GMGTs: glycerol monoalkyl glycerol tetraethers
GTDB: Genome Taxonomy Database
IMNGS: Integrated Microbial Next Generation Sequencing
JGI: Joint Genome Institute
MES: morpholinoethanesulfonic acid monohydrate
ML: maximum likelihood
NCBI: National Center for Biotechnology Information
