# Supplementary material for: Methanosuratincola petrocarbonis gen. nov., sp. nov., a methyl-reducing methanogen isolated from Shengli oil field, and proposal of Methanosuratincolaceae fam. nov., Methanosuratincolales ord. nov. and Methanosuratincolia classis nov. in the phylum Thermoproteota
Source: Int J Syst Evol Microbiol. 2025 Jul 10;75(7):006839. doi: 10.1099/ijsem.0.006839 (PMC12244365; doi:10.1099/ijsem.0.006839)
Supplement: Uncited Supplementary Material 1. [file ijsem-75-06839-s001.pdf]

Supplementary material for

Methanosuratincola petrocarbonis gen. nov., sp. nov., a methyl-reducing methanogen isolated from Shengli oil field, and proposal of *Methanosuratincolaceae* fam. nov., *Methanosuratincolales* ord. nov., and *Methanosuratincolia* classis nov. in the phylum *Thermoproteota*

Authors

Kejia Wu[#1,2], Lei Zhou[#2], Fengfeng Zheng[3], Laiyan Liu[2], Min Yang[2], Jiang Li[2], Diana Z. Sousa[*1], Lei Cheng[*2]

Affiliations

[1], Laboratory of Microbiology, Wageningen University and Research, Wageningen, The Netherlands

[2], Key Laboratory of Development and Application of Rural Renewable Energy, Biogas Institute of Ministry of Agriculture and Rural Affairs, Chengdu, China

[3], Shenzhen Key Laboratory of Marine Geo-Omics Research, Southern University of Science and Technology, Shenzhen, China

[#], These authors contributed equally to this work

[*]**Corresponding authors**

Lei Cheng, chenglei@caas.cn, ORCID 0000-0003-1178-8190

Diana Z. Sousa, diana.sousa@wur.nl, ORCID 0000-0003-3569-1545

Supplementary Table 1 The substrate utilization of strain LWZ-6^T.

Substrate	Growth
H_2 + methanol	+
H_2 + monomethylamine	+
H_2 + dimethylamine	-
H_2 + trimethylamine	-
H_2 + methanethiol	-
H_2 + betaine	-
H_2 + CO_2	-
Formate + methanol	-
Formate + monomethylamine	-
Lactate + methanol	-
Acetate	-
Pyruvate	-
Lactate	-
Glucose	-
Casamino acids	-

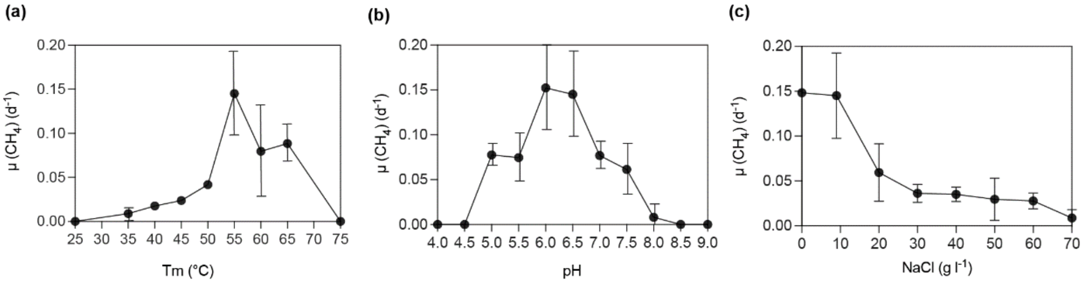

Supplementary Fig. S1. Influence of operational parameters on methane production by strain LWZ-6^T. (a) Temperature effects (tested at pH 6.5 and 9 g l^{-1} NaCl); (b) pH effects (tested at 55 °C and 9 g l^{-1} NaCl); (c) NaCl concentration effects (tested at 55 °C and pH 6.5). μ represents the maximum specific methane production rate.

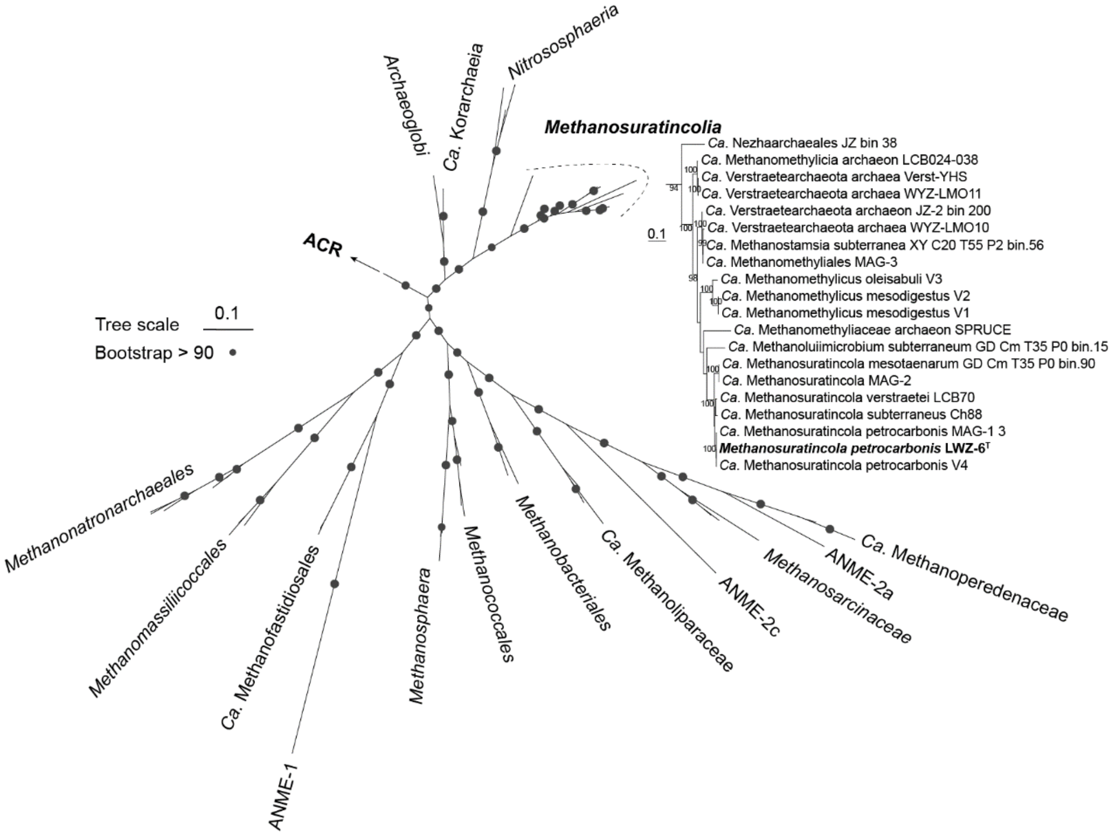

Supplementary Fig. S2. Phylogeny tree based on *mcrA* gene. The tree was generated using IQ-TREE (LG+C40+F+G model) with 1,000 ultrafast bootstraps.
